# Factors Affecting Inpatient Mortality in Elderly People with Acute Kidney Injury

**DOI:** 10.1155/2018/2142519

**Published:** 2018-05-06

**Authors:** Muhammad Abdul Mabood Khalil, Safia Awan, Rabeea Azmat, Muhammad Ashhad Ullah Khalil, Nazish Naseer, Jackson Tan

**Affiliations:** ^1^Aga Khan University Hospital, Karachi, Pakistan; ^2^Khyber Teaching Hospital Peshawar, Khyber Pakhtunkhwa, Pakistan; ^3^RIPAS Hospital, Bandar Seri Begawan, Brunei Darussalam

## Abstract

**Background:**

Acute Kidney Injury (AKI) is common in elderly people (EP). There is paucity of data on predictor of mortality in EP with AKI.

**Objective:**

This study was done to know more about factors associated with inpatient mortality in EP with AKI.

**Methods:**

We retrospectively reviewed medical records of patients aged 65 years or above hospitalized with a diagnosis of AKI at Aga Khan University Hospital, Karachi, between January 2005 and December 2010. Binary logistic regression models were constructed to identify factors associated with mortality in EP with AKI.

**Results:**

431 patients had AKI, with 341 (79.1%) having stage I AKI, 56 (13%) having stage II AKI, and 34 (7.9%) having stage III AKI. Out of 431 patients, 142 (32.9%) died. Mortality increased with increasing severity of AKI. Mortality was 50% (17/34) in AKI stage III, 44.6% (25/56) in AKI stage II, and 29.3% (100/341) in AKI stage I. Factors associated with increased inpatients mortality were presence of stage III AKI (OR: 3.20, *P* = 0.04, 95% CI: 1.05–9.72), presence of oliguria (OR: 3.42, *P* = 0.006, 95% CI: 1.42–8.22), and need for vasopressors (OR: 6.90, *P* < 0.001, 95% CI: 2.42–19.65). Median bicarbonate 18 versus 17 between those who survived and those who died was associated with less mortality (OR: 0.94, *P* = 0.02, 95% CI: 0.89–0.99). History of hypertension (OR: 0.49, *P* = 0.03, 95% CI: 0.25–0.95) and high admission creatinine (OR: 0.68, *P* = 0.01, 95% CI: 0.50–0.91) were also associated with less mortality.

**Conclusion:**

Mortality in EP increases with increasing severity of AKI. Presence of stage III AKI, oliguria, and hemodynamic instability needing vasopressor are associated with increased mortality. Increased median bicarbonate, presence of hypertension, and high admission creatinine were various factors associated with decreased inpatient mortality. Increasing age and need for dialysis did not increase mortality in elderly population.

## 1. Introduction

EP are prone to develop AKI due to comorbid conditions and age-related changes in the kidney. There is age-related glomerulosclerosis, mesangial expansion, thickening of glomerular basement membrane, and decrease in the number of tubules, resulting in reduced renal mass [1–4]. The combination of age-related changes and presence of comorbid conditions makes EP prone to develop AKI when exposed to any acute health-related issues. The incidence of AKI is increasing worldwide [5–7] with increased incidence in EP [7]. AKI causes morbidity, mortality, and significant cost to healthcare system. Mortality of AKI in EP varies from 20 to 45% as reported in various studies [7–9]. There is little work done on factors associated with mortality in AKI in EP. Identification of factors associated with mortality may identify risk factors, which when present can lead to worse outcome. Manipulating these factors may improve mortality further in the future. With this in mind, this study was designed to investigate and identify potential factors that may determine mortality in this specific group of patients.

## 2. Materials and Methods

This retrospective study was conducted at Aga Khan University Hospital, Karachi, Pakistan. Aga Khan University Hospital is a tertiary healthcare facility located in the biggest city in Pakistan. It has 15 inpatient units with a total capacity of 563 beds and is also the tertiary referral center for advanced diagnostic procedures and management. This study was conducted at the Department of Medicine.

Data were collected from the start of 2005 to 2010 for a period of 5 years after receiving approval from the ethics review committee of the institution. All patients consented on admission for publication of their data for research purposes as per hospital policy. Study population included EP with AKI. EP were defined as population having age of 65 years or above [10, 11]. EP with AKI and age of 65 or above were included in the study. Patients with history of chronic kidney disease and age below 65 years were excluded. Patients who have no reference creatinine in the past 3 months were also excluded. Data on demographics, clinical features, laboratory investigations, and mortality were noted on a proforma.

Records of the cases were retrieved through codes using international classification of disease (ICD). The ICD code for AKI is 584.9. All the files were retrieved through identification of ICD code for AKI. AKI was defined as any of the following according to KDIGO guidelines [12]:

- Increase in serum creatinine by ≥0.3 mg/dl (≥26.5 micromole/l) within 48 hours
- Increase in serum creatinine to ≥1.5 times compared to baseline, which is known or presumed to have occurred within the prior 7 days
- Urine volume <0.5 ml/kg/h for 6 hours

AKI was further classified into the following stages:

**Stage 1 .**

Increase in serum creatinine 1.5–1.9 times compared to baseline or ≥0.3 mg/dl (≥26.5 mmol/l) increase and urine output <0.5 ml/kg/h for 6–12 hours.

**Stage 2 .**

Increase in serum creatinine 2.0–2.9 times compared to baseline and urine output <0.5 ml/kg/h for ≥12 hours.

**Stage 3 .**

Increase in serum creatinine 3.0 times compared to baseline or increase in serum creatinine to ≥4.0 mg/dl (≥353.6 *μ*mol/l) or initiation of renal replacement therapy or, in patients below 18 years of age, decrease in eGFR to <35 ml/min per 1.73 m^2^ or anuria for ≥12 hours.

EP were defined as population having age of 65 years or above [10, 11]. The cohort was assessed for factors associated with inpatient mortality by dividing it into two groups. One group included patients who survived and the other group included patients who died. Mortality comparison for ages of 65–80 years and >80 years was also done. Ethical approval was taken from both departmental and hospital ethics review committee. All patients consented on admission for publication of their data for research purposes in the future as per hospital policy.

## 3. Statistics

A descriptive analysis was done for demographic, clinical, and radiographic features and results are presented as mean ± standard deviation for quantitative variables and number (percentage) for qualitative variables. Differences in proportions were assessed by using the Chi-square test or Fisher's exact test where appropriate. For contrasts of continuous variables, independent sample *t*-test was used to assess the difference of means. Multivariable analysis was conducted to identify the factors of in-hospital mortality in AKI patients.

All analyses were conducted by using the Statistical Package for Social Sciences (SPSS) (release 16.0, standard version, copyright © SPSS; 1989-02).

## 4. Results

476 patients were analyzed. 30 patients having chronic kidney diseases were excluded. Another 15 patients having no baseline creatinine in previous three months were also excluded. At the end, 431 patients who had AKI and who satisfied our inclusion criteria were analyzed. 431 patients had AKI, with 341 (79.1%) having stage I AKI, 56 (13%) having stage II AKI, and 34 (7.9%) having stage III AKI. Urine routine examination was available for 411 patients. It showed pyuria in 11 and proteinuria or hematuria in 48 and was bland in 352. Kidney biopsy was performed in 44 cases, showing acute tubular necrosis in 8, glomerulonephritis or vasculitis in 24, and interstitial nephritis in 12. 348 (80.7%) were aged 65–80 years. 83 patients (19.3%) were aged above 80 years. Out of 431 patients, 250 (58%) were males and 181 (42%) were females. We analyzed various factors that could affect mortality. These included age, comorbid conditions like diabetes and hypertension, admission creatinine, peak creatinine, pH, O_2_, CO_2_, HCO_3_, albumin, presence of oliguria, need for vasopressor, stay in ICU, and severity of AKI. On univariate analysis, hypertension (*P* = 0.055), admission creatinine (*P* = 0.02), peak creatinine (*P* = 0.054), AKI stage III (*P* = 0.009), need for ventilation (*P* = 0.01), need for vasopressor (*P* < 0.001), need for ICU (*P* = 0.004), and presence of oliguria (*P* = 0.001) were found to be associated with increased mortality (Table 1). 142 patients (32.9%) died. Mortality varied proportionally with severity of AKI. 144 patients (32.9%) died. Mortality was 50% (17/34) in stage III AKI, 42.9% (24/56) in stage II AKI, and 29.03% (99/341) in stage III AKI (Table 1).

However, on multivariate logistic regression analysis, factors associated with increased inpatients mortality (Table 3) included presence of stage III AKI (OR: 3.20, *P* = 0.04, 95% CI: 1.05–9.72), presence of oliguria (OR: 3.42, *P* = 0.006, 95% CI: 1.42–8.22), and need for vasopressors (OR: 6.90, *P* < 0.001, 95% CI: 2.42–19.65). High median bicarbonate (OR: 0.94, *P* = 0.02, 95% CI: 0.89–0.99), history of hypertension (OR: 0.49, *P* = 0.03, 95% CI: 0.25–0.95), and high admission creatinine (OR: 0.68, *P* = 0.01, 95% CI: 0.50–0.91) were associated with lesser mortality. Significant number of our patients have low albumin. Interestingly, unlike chronic kidney disease, hypoalbuminemia in our cohort did not contribute to increased mortality. Similarly, the presence of diabetes did not contribute to mortality in our cohort. Increasing age (65–80 versus >80 years) did not have any impact on mortality. Furthermore, the need for hemodialysis was not associated with increased mortality in our cohort. At the same time, there were no survival benefits with use of hemodialysis (11 died and 9 survived). 20 patients needed dialysis. 15 patients were treated with intermittent hemodialysis and 5 received continuous renal replacement therapy. 6 patients needed 10 sessions, 2 patients needed 9 sessions, and 5 patients needed 8 sessions. 2 patients needed three times per week upon discharge. 11 patients needing renal replacement therapy survived in comparison to 9 patients who died. 6 patients needing renal replacement therapy recovered full renal function. 3 had partial recovery with no need for dialysis. 2 patients developed stage 5 chronic kidney disease and were dialysis-dependent till 6 months of follow-up (Table 2).

## 5. Discussion

AKI is common worldwide. Incidence rates have been reported in many studies varying within an average of 23.8 cases per 1000 discharges [6, 13, 14] and there is 11% yearly increase between 1991 and 2001 [6]. Like other age groups, EP are also more prone to develop AKI due to multiple comorbid conditions and age-related changes in the kidney. As a result, the incidence of AKI has increased significantly in EP [7]. AKI has been associated with significant morbidity, mortality, and enormous cost to the health system.

Inpatient hospital mortality in patients with AKI has been reported to be 10.8%–42.8% in various studies [7, 9, 15–17]. We found mortality of around 32.9%, which is comparable to the results found in other studies. Mortality in AKI is highly variable due to a variety of reasons. It can be low in community-acquired AKI (19.6%) as compared to hospital-acquired AKI (42.8%) [16]. Similarly, high mortality ranging from 66% to 71.5% has been reported in patients admitted to ICU with AKI [13, 18]. Mortality is even worse (81%) if AKI develops after 7 days of ICU admission [18]. AKI due to sepsis has very high mortality of 74% when compared to nonseptic AKI, where mortality is 45% [19]. Our mortality was 32.9%, which is comparable to reported 10.8%–42.8% internationally [7, 9, 15–17]. However, it is lower than ICU, hospital-acquired, and sepsis-related AKI. This might be because we included community-acquired AKI and only few (5.6%) patients of our cohorts were from ICU. Moreover, a bulk of our patients (79.1%) were having mild stage I AKI.

For every disease, it is important to know its prognostic factors that can affect the outcome. A couple of studies are available to look for prognostic factors for mortality in patients with AKI [20–22]. However, all these studies are done in various age group patients including elderly patients. Prerenal AKI is usually due to decrease in effective circulation, which if not corrected leads to acute tubular necrosis. Theoretically, the chances of conversion from prerenal failure to intrinsic renal failure are more in elderly patients due to the presence of comorbid conditions and age-related changes. We found that the use of vasopressors in EP with AKI has propensity to die. Korula et al. also found the use of vasopressor to be associated with increased 28-day mortality [22]. Similarly, Uchino et al. [23] also found that use of vasopressor increases odds of mortality. Hypotension in event of AKI led to increased mortality in various studies [21, 24, 25]. We also found that low blood pressure during event of AKI can lead to increased mortality. Presence of hypertension in our cohort exerted a beneficial effect on mortality. We assume that this could be due to better perfusion of the kidney. However, this warrants further investigations in prospective studies. Patients with AKI and having low blood pressure due to volume depletion can benefit from fluid resuscitation to achieve good mean arterial pressure. This will have beneficial outcome on mortality. We found the need for ICU and mechanical ventilation to be significantly associated with increased mortality on univariate analysis. However, these facts were not reproduced when analyzed through logistic regression analysis. This may be due to the fact that very small proportion of our cohort needed ICU care. We found increased mortality with increasing severity of AKI. In fact, mortality was 50% in stage III AKI in our cohort.

Monitoring urine output is important to identify patients at risk of AKI as changes in creatinine may take time to appear. Presence of oliguria has got worst prognostic implications. We found that oliguria in EP with AKI was significantly associated with increased mortality. Oliguria has been found by other colleagues as a predictor of mortality [19, 21, 24, 26]. Oliguria and abnormal creatinine when used together have been shown to predict the highest mortality as shown in a cohort of 32,000 patients [14]. AKI causes various complications including metabolic acidosis. We found that the median bicarbonate in those who survived was high as compared to those who died. High median bicarbonate has a beneficial effect on reducing mortality. We found high admission creatinine to be associated with less morality. This could be due to earlier alertness of clinician to start goal-directed therapy. Alternatively this may point to more community acquired AKI in our cohort, which has better prognosis. This finding needs further assessment preferably through prospective studies.

Although diabetes was also present in a significant number of patients in our cohort, analysis of our data did not show any impact of diabetes on mortality. Low albumin has been shown to increase mortality in chronic kidney disease patients [27, 28]. However, we failed to show that low albumin leads to increased mortality in EP with AKI in our study.

The studies we cited so far for mortality and its predictors included all age groups from young to elderly. None of them were specifically done on elderly population. Various prognostic factors we found which could relate to mortality in the elderly were also reported for other age groups as discussed. One may think that, in presence of age-related changes, EP may do worse. However, we found interesting trend that increasing age within elderly populations was not associated with worse outcome. The difference in mortality in our cohort with increasing age (65–80 years versus >80 years) was not significant. Rather the mortality of 32.9% was comparable to other age groups, where mortality ranged from 10.8% to 42.8% [7, 9, 15–17]. Our literature search showed that only few studies are done in elderly populations. Van Den Noortgate et al. did a retrospective analysis of postcardiac surgery AKI in EP who needed dialysis. They showed similar outcome between elderly and young populations in terms of mortality [29]. In our study, we found a similar trend and need for hemodialysis did not have any impact on inpatient mortality. The absence of a relationship between age and poor outcome in the elderly developing AKI in the intensive care unit has already been confirmed in another study [30]. Pasciial and Llaño [8] analyzed mortality in EP with AKI. They compared age above 80 years, age of 65–79 years, and age below 65 years. They found no difference in mortality across all the age groups. From this, we can assume that outcome of AKI in terms of mortality is similar to other age groups. Therefore, if EP need dialysis in an event of AKI, they should not be discriminated on the basis of age.

## 6. Strength and Limitation of the Study

The strength of this study is that it is one of the few studies in elderly Pakistani Asian population, which analyzed a large cohort of patients for prognostic factors associated with mortality. We acknowledge that there are several limitations in this study. This data is retrospective and is from a single center. We would have liked to report etiology and classification of AKI as prerenal, renal (acute tubular necrosis), and postrenal if the study was done prospectively. The definition of elderly person has been variable and as a result the findings of this study can be applied to the age groups mentioned in this study only. We also acknowledge that using code to identify AKI can lead to error and may miss some data. We admit that lack of baseline creatinine in previous 3 months could have excluded some patients with AKI from our cohort, resulting in selection bias. However, the number of such patients was only 15 and we could not classify them as AKI or chronic kidney disease even from the review of notes. We acknowledge that there was a lack of long-term follow-up to show the long-term effect of AKI on morbidity and mortality in EP.

## 7. Conclusion

We conclude from this study that mortality in EP increases with increasing severity of AKI. Severe AKI (stage III), oliguria, and hemodynamic instability needing vasopressor were associated with increased mortality. Presence of hypertension, high median carbonate, and high admission creatinine were associated with beneficial effect on mortality. Mortality of AKI in EP is comparable to other studies. Moreover, increasing age within elderly population is not associated with worse outcome. Age is not a particularly poor prognostic sign, and outcome seems to be acceptable for very old patients with AKI. Therefore, EP with above risk factors should get meticulous resuscitation and renal support under close monitoring. Furthermore, acute dialysis should not be withheld from patients solely because of their increasing age.

## Figures and Tables

**Table 1:** Descriptive characteristics of study population (*n* = 431).

	Total	Survived, *n* = 289 (67.1%)	Died, *n* = 142 (32.9%)	*P* value
Age				
65–80 years	348 (80.7%)	231 (79.9%)	117 (82.4%)	0.61
>80 years	83 (19.3%)	58 (20.1%)	25 (17.6%)	
Length of stay, days, median [IQR]	4 [2–8]			
Diabetes	133 (30.9%)	94 (70.7%)	39 (29.3%)	0.32
Hypertension	228 (55.9%)	163 (71.5%)	65 (28.5%)	0.055
Admission Cr, median [IQR]	2.1 [1.4–3.1]	2.2 [1.5–3.3]	2 [1.2–2.8]	0.02^*∗*^
Peak Cr	2.8 [2.1–4.1]	2.7 [2–4]	3 [2.2–4.1]	0.054^*∗*^
Discharge Cr	1.8 [1.3–2.9]	1.5 [1.1–2.2]	2.7 [1.8–3.6]	<0.001^*∗*^
pH	7.3 ± 0.29	7.3 ± 0.36	7.3 ± 0.13	0.99
O_2_	81.6 ± 21.7	81.6 ± 21.2	81.5 ± 22.7	0.98
pCO*₂*	34 ± 15.2	33.9 ± 15.2	15.3 ± 1.6	0.95
Albumin, *n* = 280				
<3.5	260 (92.9%)	162 (91.5%)	98 (95.1%)	0.33
>3.5	20 (7.1%)	15 (8.5%)	5 (4.9%)	
HCO_3_, *n* = 224				
<21	156 (69.6)	92 (68.1)	64 (71.9)	0.65
>21	68 (30.4)	43 (31.9)	25 (28.1)	
HCO_3_, median [IQR]	18 [13–23]	18 [15–23]	17 [12–22]	0.12^*∗*^
Need for dialysis	20 (4.5)	11 (3.7)	9 (6.2)	0.23
Ventilation	32 (7.2)	15 (5)	17 (11.6)	0.01
Vasopressor	56 (12.6)	15 (5)	41 (28.1)	<0.001
Stay in ICU	25 (5.6)	10 (3.3)	15 (10.3)	0.004
Oliguria	63 (14.2)	31 (10.4)	32 (21.9)	0.001
AKI stage 1	341 (79.1%)	241 (70.7%)	100 (29.3%)	NS
AKI stage 2	56 (13%)	31 (55.4%)	25 (44.6%)	NS
AKI stage 3	34 (7.9%)	17 (50%)	17 (50%)	NS

*Outcome*				
Survived	299 (67.1)			
Died	146 (32.9)			

**Table 2:** Stages, mode of renal replacement therapy, outcome of patient on renal replacement therapy, and inpatient mortality in patients with AKI.

Stages of AKI		Mode of renal replacement therapy	Outcome of patients needing renal replacement therapy	Inpatient mortality
Stage I AKI Stage II AKI Stage III AKI	341 (79.1%) 56 (13%) 34 (7.9%)	Intermittent hemodialysis = 15 patients Continuous renal replacement therapy = 5	Patients needing renal replacement therapy = 20 Died = 9 Survived = 11 Full recovery = 6 Partial recovery = 3 Chronic kidney disease needing dialysis = 2	Survived = 299 (67.1%) Died = 146 (32.9%)

**Table 3:** Multivariable analysis of factor predicting outcome (mortality) in AKI patients.

	Odds ratio [95% CI]	*P* value
*Factors predicting worse outcome*		
Oliguria		
No	1.0	0.006
Yes	3.42 [1.42–8.22]	
Vasopressor		
No	1.0	<0.001
Yes	6.90 [2.42–19.65]	
AKI stage III versus AKI stages I and II	1.0	0.04
	3.20 [1.05–9.72]	

*Factors predicting better outcome*		
HTN		
No	1.0	0.03
Yes	0.49 [0.25–0.95]	
Admission Cr	0.68 [0.50–0.91]	0.01
Median bicarbonate	0.94 [0.89–0.99]	0.02

*Factors having no effect on the outcome*		
Peak Cr	1.04 [0.87–1.25]	0.63
Albumin	0.66 [0.41–1.06]	0.08
DM		
No	1.0	0.51
Yes	0.78 [0.37–1.63]	

## Abbreviations

AKI: Acute Kidney Injury
EP: Elderly people.
